# Molecular typing, biofilm production, and detection of carbapenemase genes in multidrug-resistant *Acinetobacter baumannii* isolated from different infection sites using ERIC-PCR in Hamadan, west of Iran

**DOI:** 10.1186/s40360-021-00504-y

**Published:** 2021-06-08

**Authors:** Maryam Hazhirkamal, Omid Zarei, Mahsa Movahedi, Pezhman Karami, Leili Shokoohizadeh, Mohammad Taheri

**Affiliations:** 1 Student Research Committee, Hamadan University of Medical Sciences, Hamadan, Iran; 2 Department of Microbiology, Faculty of Medicine, Hamadan University of Medical Sciences, Hamadan, Iran

**Keywords:** Molecular typing, Biofilm formation, MBL, MDR, *Acinetobacter baumannii*

## Abstract

**Background:**

*Acinetobacter baumannii* is an opportunistic pathogen that can cause several kinds of nosocomial infections. Increasing antibiotic resistance as well as identifying genetic diversity and factors associated with pathogenicity and prevalence of this bacterium is important. The aim of this study was the investigation of molecular typing, biofilm production, and detection of carbapenemase genes in multidrug-resistant *Acinetobacter baumannii* isolated from different infection sites using ERIC-PCR in Iran.

**Methods:**

Forty isolates of *A. baumannii* were obtained from various wards of the central hospital, in the west of Iran. Phenotypic identification and genetic diversity, biofilm production assay, and detection of Carbapenemase genes carried out.

**Results:**

Tracheal samples 26 (61.9 %) are the most frequent isolates, and 95 % of isolates were identified as MDR. 32.5 % of all *A. baumannii* strains were capable to form a strong biofilm. It was founded that antimicrobial resistance patterns had a significant relationship with strong biofilm formation (*P* = 0.001). Most frequencies of the studied genes were in the order of *VIM* (81 %), *SPM* (45.2 %), and *IMP* (35.7 %) genes. The *VIM* gene was the most frequent in all isolates which were significant (*P* = 0.006). 14 different ERIC-types were observed including 7 common types and 7 unique or single types. F type is the largest common type consisting of nine isolates and B, D, and E types contain two isolates separately.

**Conclusions:**

ERIC-PCR technique was used to genetically classify *A. baumannii* isolates as one of the most common microorganisms in nosocomial infections.

## Background

*Acinetobacter baumannii* is an opportunistic pathogen that can cause several kinds of nosocomial infections, including surgical site infections, skin and soft tissue infections, urinary tract infections (UTI), ventilator-and nosocomial‑associated pneumonia, catheter-related bloodstream infections, and secondary meningitis [1, 2]. This bacterium is unique due to enabling it to survive in a variety of conditions for long periods and to colonize on the surfaces and materials in hospitals, enabling its transfer between patients through human reservoirs or inanimate elements [3, 4]. Besides, this organism exhibits resistance against several types of antibiotics [4]. In light of recent concerns about multidrug-resistant (MDR) and extensively drug-resistant (XDR) isolates, now infections caused by *A. baumannii* present a public health issue worldwide [5].

In the last decade, carbapenems have been suggested as a choice drug for treating serious infections related to MDR isolates of Acinetobacter [6]. Metallobetalactamase enzymes, which can hydrolyze almost all beta-lactams except monobactams, are one of the most common carbapenem resistance mechanisms. Moreover, genes encoding Metallo-β-lactamases that are placed on integrons can be transferred from one bacterium to another one easily via plasmids [7]. The *IMP*, *VIM*, and *SPM* genes are related to Metallo-β-lactamases producers in Acinetobacter that cause resistance to many antimicrobial drugs [8]. Recently, many studies have been emphasized the biofilm formation in *A. baumannii*, because microbes that present in the biofilm structure have less sensitivity to antimicrobials and are more resistant to environmental situation surfaces such as catheters, intubation tubes, and cleaning types of equipment, which indicates the critical role of biofilm in the treatment failure and increase hospitalization time [9, 10].

The study of molecular epidemiological characteristics could help in the management of bacterial spread in hospitals and *A. baumannii* drug resistance [2, 5, 11]. Several important genotypic methods have been introduced, including pulsed-field gel electrophoresis (PFGE) and multilocus sequence typing (MLST), which are used to detect the genotypic relationship of *A. baumannii* strains in hospital settings. Furthermore, PCR-based methods, including enterobacterial repetitive intergenic consensus (ERIC)-PCR, are valuable typing methods for non-fermentative gram-negative bacilli [12, 13]. Molecular typing has a pivotal role in understanding the essential mechanisms of *A. baumannii* infections and discovering the relationship between bacterial species. Overall, evidence suggests that molecular typing techniques are now an effective method for assessing and defining the primary cause of infection in hospitals [14]. Genotype techniques such as PFGE, MLST, and ERIC-PCR can be used as typing methods in microorganisms, especially bacteria, due to their availability, cost-effectiveness and ability to be done in less time. The ERIC-PCR is a molecular technique used in epidemiological and genotyping studies of bacteria. This method provides great potential to study bacterial sequences because the sequences are longer and do not base on a specific region of the genome. This approach also provides detailed information for researching and comparing the genomes of a wide variety of bacterial species [15, 16]. The ERIC PCR method can be used to study human pathogens to determine their genetic diversity [17].

The purpose of this study is to investigate the relationship between molecular typing, biofilm formation, and detection of carbapenemase genes in multidrug-resistant *A. baumannii* isolated from different infections in Hamadan, Iran.

## Materials and methods

### Sample collection

To begin, all subjects signed a consent form, and all procedures were carried out in compliance with Hamadan’s ethics committee guidelines. Forty, *A. baumannii* isolates were obtained from hospitalized patients in different wards in Hamadan, Iran, were collected from August 2019 to September 2020. To confirm isolates, morphological and biochemical tests including the API method were performed.

### Antimicrobial susceptibility testing

The antimicrobial susceptibility test was determined on the Mueller–Hinton agar (Merck, Germany) using the Kirby Bauer (disc diffusion) method as CLSI 2018 guidelines [18]. The antibiogram test was performed using the antibiotics including Amikacin (30 µg), levofloxacin (5 µg), co-trimoxazole (1.25/23.75 µg), Ceftazidime (30 µg), piperacillin-tazobactam (100 µg/10 µg), Imipenem (10 µg) ampicillin-sulbactam (10 µg/10 µg), (10 µg), meropenem (10 µg),. The standard bacterial strain of *E.coli* (ATCC 25,922) was used as quality control. (MAST, Group Ltd., Merseyside, UK). *A. baumannii* isolates when they are non-susceptible to at least one agent in three or more antimicrobial categories were considered as multidrug-resistant (MDR) [19].

### Biofilm Assay

The *A. baumannii* isolates were evaluated for their ability to biofilm formation using microplate. A 0.5 McFarland standard turbidity of *A. baumannii* suspension was prepared; 200 µL of suspension was added to each well and incubated overnight at 35 °C. The wells were washed three times with normal saline, then to fix cells, 200 µL ethanol (96 %) was added to wells; the good contents were pulled after 15 min, and let’s plate was dried at room temperature. The staining step was carried out by adding 200 µL of 2 % crystal violet for 5 min. After the color removal, 200 µL of 33 % acetic acid was added and incubated at 37 °C for 15 min. Empty wells containing media were considered as a negative control. The absorbance of each well was recorded at 560 nm using an ELISA reader. For each sample, the biofilm assay was repeated three times, and data were measured.

### DNA Extraction and detection of carbapenemase genes

The isolates were cultured in Luria Bertani (LB) broth and incubated at 35 °C overnight. DNA extraction was performed via the boiling method. The DNA concentration was determined using nanodrop. The PCR reactions were prepared in 25 µl total volume as follow 10 µl of Taq DNA Polymerase, Master Mix (Amplicon, Denmark), with 1 µl (50 ng) of extracted DNA, 1 µl for forward and reverse primers, and sterile deionized water to get a final volume of 25 µl. The amplifications were carried out in a thermocycler (Bio-Rad, USA), with the following conditions: initial denaturation at 95 °C for 1 min, followed by 35 cycles of denaturation at 95 °C, for 30 s, annealing 53 °C for 40 s, and extension at 72 °C for 90 s with a final extension at 72 °C for 4 min (Table 1).

**Table 1:** Primers for identification of carbapenemase-producing bacteria

Primer		Size bp	Reference
**VIM-Family**	F: GATGGTGTTTGGTCGCATA R: CGAATGCGCAGCACCAG	390	(Ellington et al., 2006)
**IMP-Family**	F: GGAATAGAGTGGCTTAAYTCTC R: CCAAACYACTASGTTATC	188	(Ellington et al., 2006)
**SPM-1**	F: AAAATCTGGGTACGCAAACG R: ACATTATCCGCTGGAACAGG	271	(Ellington et al., 2006)

### Molecular typing and ERIC-PCR

**Fig. 1:**
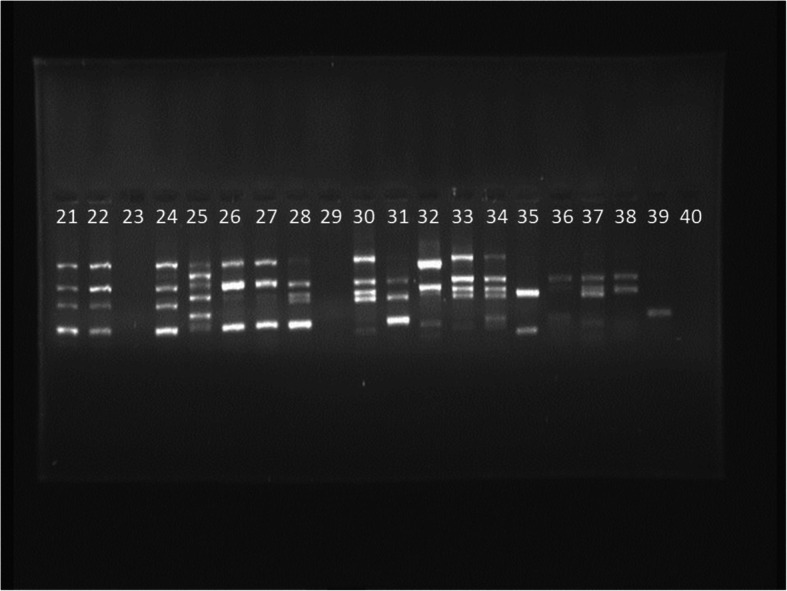
Agarose gel electrophoresis of ERIC-PCR products of *A. baumannii* isolates

In this method, PCR in a reaction with a volume of 50 µl including 5 µl of PCR buffer10x, 4 µl Mgcl2, 75/1 µl dNTP Mix, 6 µl of Taq DNA Polymerase enzyme, 3 µl of primer pairs ERIC1: ATGTAAGCTCCTGGGGATTCAC and ERIC2: AAGTAAGTGACTGGGGTGAGCG. ERIC-PCR test was performed on each of the isolates 3 times. NTSYS version 2.02e software was used to draw the phylogenetic tree and analyze the images obtained by electrophoresis of the studied samples (Fig. 1). The bands obtained from the electrophoresis of the marker product were scored as quantitative data of zero and one (presence or absence of band). After scoring the gels, the genetic similarity was calculated based on data zero and one using Jaccard and Dice coefficient and simple matching. To determine the efficiency of the ERIC method of cluster analysis based on similarity coefficients, the coefficient of correlation coefficient was used. Then, to group the strains, cluster analysis by method Unweighted Pair Group Method using arithmetic Averages (UPGMA) was used based on the similarity coefficient that had the highest coefficient of coronary correlation.

### Statistical analysis

The t-test was used to compare categorical results. All statistical tests were two-tailed, and statistical significance was defined as a P-value of 0.05. The statistical software package SPSS version 22 (IBM, NY) was used to analyze the data.

## Results

### Bacterial isolates and antimicrobial susceptibility test

In this study, 40 non-duplicated *A. baumannii* isolates were obtained from the various clinical samples, including wounds 5 (11.9 %), CSF 2 (4.8 %) tracheal samples 26 (61.9 %), blood 3 (7.1 %), and urine 4 (9.5 %) isolates and the mean age of the patients was 33.5 ± 22.2 years.

The frequency samples collected from males and females were 23 (57.5 %) and 17 (42.5 %), respectively in this study. According to antibiotic susceptibility testing, 38 out of 40 (95 %) *A. baumannii* isolates were identified as MDR. In our study, among 38 MDR *A. baumannii* isolates, were resistant to Ampicillin/Sulbactam 36 (90 %), Amikacin 38 (95 %), Ceftazidime 38 (95 %), Imipenem 39 (97/5 %), Meropenem 38 (95 %), Levofloxacin 36 (90 %), Piperacillin/Tazobactam 39 (97/5 %), Trimethoprim/Sulfamethoxazole 36 (90 %). In addition, all isolates were susceptible to Colistin.

### Biofilm formation

Out of 40 *A. baumannii* isolates, 13 (32.5 %) of all *A. baumannii* strains produced a strong biofilm, while 10 (25 %) and 17 (42.5 %) of these isolates were considered as weak and medium biofilm-producer isolates, respectively. Also, this study showed a significant relation of antimicrobial resistance patterns with strong biofilm production (*P* = 0.001). High strong biofilm formation in different clusters showed in Table 2. No significant relationship was observed between biofilm production and clusters.

**Table 2:** High frequency genes and Strong biofilm formation in different clusters

Cluster	A	B	C	D	E	F	G	H
**Frequency**	6 (15 %)	2 (5 %)	5 (12/5 %)	2 (5 %)	2 (5 %)	2 (5 %)	9 (22/5 %)	4 (10 %)
**High frequency gene**	VIM (14.7)	VIM (5.88)	SPM (15.78)	IMP (6.66)	IMP (13.33)	VIM (2.94)	SPM (36.84)	IMP (20)
**Strong biofilm formation**	2 (15.38)	1 (7.69)	4 ( 30.7)	1 (7.69)	1 (7.69)	1 (7.69)	2 (15.38)	1 (7.69)

### Detection of carbapenemase genes

**Fig. 2:**
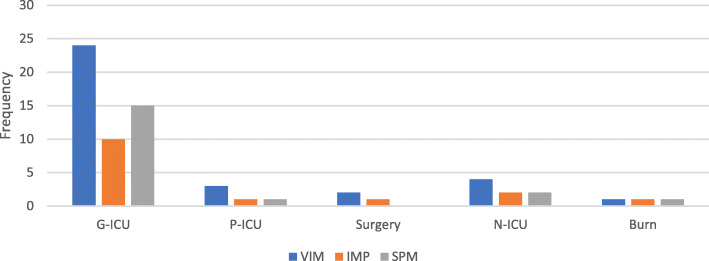
Frequnces of MBL-producing genes in different wards

The frequencies of the studied genes are in order of VIM (81 %), SPM (45.2 %), and IMP (35.7 %) genes. The frequency of carbapenemase encoding genes in different sources is presented in Fig. 2. High-frequency genes in different clusters are presented in Table 2. The *VIM* gene was the most frequent in all isolates significantly (*P* = 0.006).

### ERIC-PCR results

**Fig. 3:**
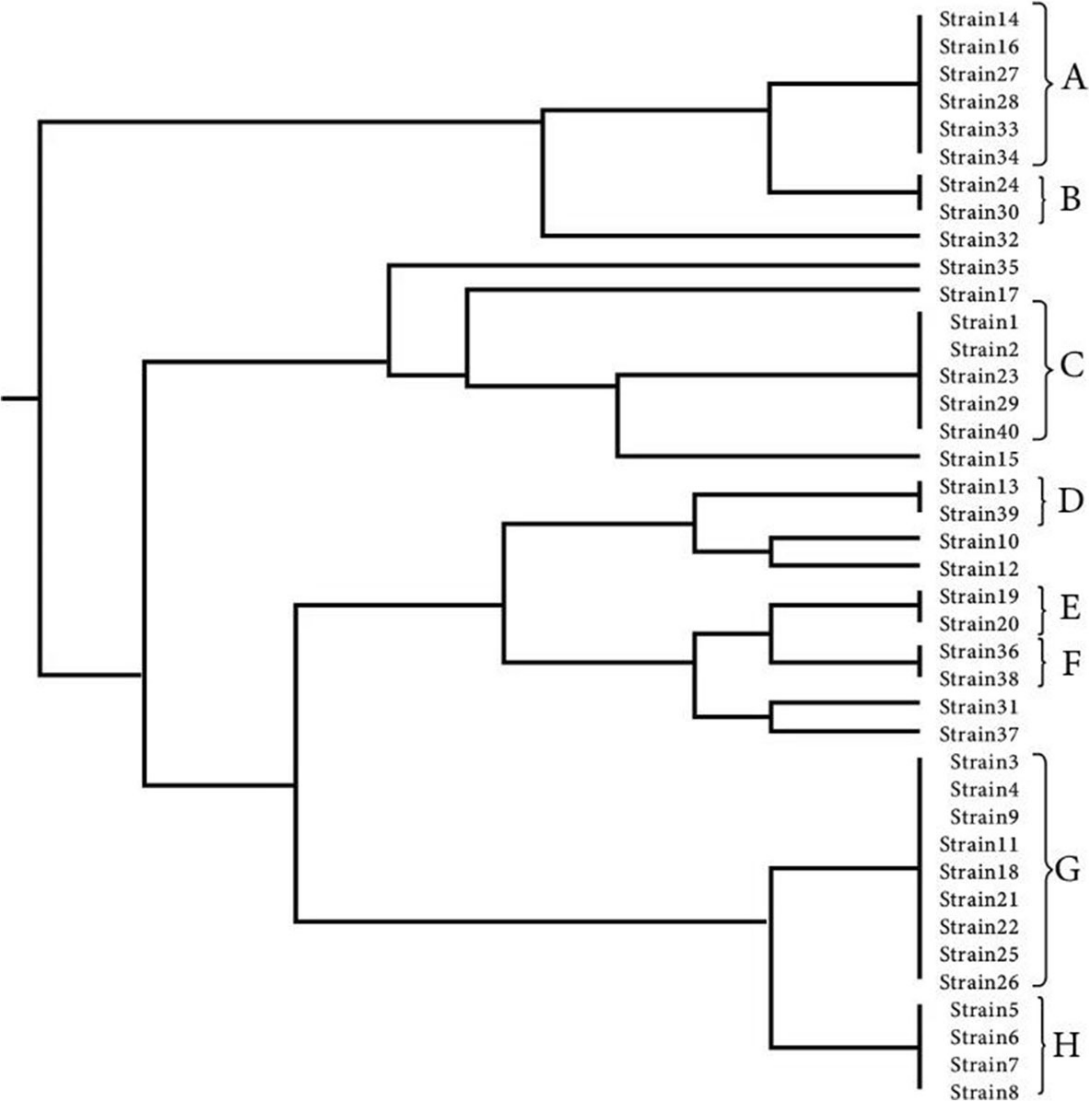
Dendrogram of *A. baumannii* isolates clustering based on ERIC patterns

ERIC-PCR results were analyzed using online software (http://insilico.ehu.es). The one to six ERIC band patterns were observed. A dendrogram was drawn (Fig. 3) and results indicated the existence of genetic diversity in *A. baumannii* isolates. According to Tables 3 and 14 different ERIC-types were observed including 7 common types, and 7 unique or single types (strains No. 10, 12, 15, 31, 32, 35, 3) were observed. F type is the largest common type consisting of nine isolates and B, D, and E types contain two isolates separately. No band was observed in isolates No. 1, 2, 23, 29, and 40, and these four isolates were classified into C type, which requires more accurate molecular typing techniques specifically sequencing-based methods to better identification of the isolates. Molecular typing of the analysis showed that the most frequent isolates belong to group G.

**Table 3:** Frequency of clusters in different wards of the hospital

Clusture	G-ICU	P-ICU	Surgery	N-ICU	Burn	Total
A	5	0	0	1	0	6
B	0	0	1	0	1	2
C	2	2	0	0	1	5
D	0	0	0	1	1	2
E	1	0	0	0	1	2
F	1	0	1	0	0	2
G	8	0	0	1	0	9
H	2	1	1	0	0	4
Total	19	3	3	3	4	32

## Discussion

*A. baumannii* is an important nosocomial pathogen that is highly resistant to many antibiotics especially carbapenems, has been reported in recent years [20, 21]. In our study, more than 95 % of isolated strains were resistant to antimicrobial agents except Colistin that all isolates were sensitive.

In Amin et al., study, 77 out of 85 MDR isolates were detected that only 2 isolates were resistant to Colistin which is inconsistent with a present study that all isolates were sensitive to Colistin. Also, blaVIM gene was the most common gene encoding MBL followed by blaIMP, blaSPM which is similar to our study [22]. In Ranjbar *et al., a* study in 2019, more than 93 % of *A. baumannii* samples were resistant to the studied antibiotics, but high sensitivity to Polymyxin (100 %) and Colistin (85.9 %) was observed, indicating the effect of this antibiotic on Acinetobacter [23]. In the study by Fallah et al., 82 samples of *A. baumannii* were isolated from the burn hospital and divided into 14 types of ERIC-PCR patterns, including 11 common types and 3 unique types. In this study, 77 samples were classified into 9 main genotypes [24]. On the other hand, in the current study 14 different ERIC-types including 7 common types and 7 unique or single types (Strains No. 10, 12, 15, 31, 32, 35, 3) were observed.

In the study of Zarifi et al., 80 samples of *A. baumannii* were isolated from ICU, which the highest frequency associated with trachea 92 % similar to the present study (65 %). They showed that *A. baumannii* isolates were classified into 14 classes using ERIC-PCR [25]. Alamri et al., examined 207 samples of MDR *A. baumannii*, which the most common frequency similar to our study, was isolated from ICU (52.5 %), and ERIC-PCR was performed on meropenem-resistant isolates. Out of 131 samples was examined and divided into 4 groups of similar ERIC-PCR patterns according to their genotypes [26].

It is known that *A. baumannii* bacteria can form biofilms on surfaces and equipment in hospitals such as catheters tubes especially in ICUs [27]. *A. baumannii* strains with strong biofilm formation ability have less sensitivity to environmental conditions like dehydration than poor biofilm formation ones; So, biofilm production is crucial for bacterial survival under dry conditions [28–30].

In this study, we investigated antibiotic susceptibility, biofilm production, and clonal relationships of different clinical MDR *A. baumannii* isolates. The highest frequency of strong biofilm formation was seen in clusters C (30.7 %) and A (15.38 %) which indicates the relationship between ICU and the formation of a strong biofilm in *A. baumannii*, and no significant relationship was observed between strong biofilm and clusters. Also, considering that 95 % of the samples were MDR, no significant difference was observed between antibiotic resistance and clusters. However, the presence of *VIM* gene in *A. baumannii* strains is common, there is also a significant relationship between *VIM* gene and clusters (*p* = 0.006), which indicates the association of resistance of this bacterium with different classes of antibiotics. In our study, there was no significant relationship between carbapenemase-producing genes and biofilm formation, which could be due to the limited samples. The highest frequency in our study was related to cluster G, which is isolated from G-ICU.

Results of our study showed that antimicrobial resistance in *A. baumannii* isolates from the different site of infections in a central hospital in Hamadan, Iran is dependent on several parameters, including the pattern of antibiotic administration in an area, geographical differences, and the extent to which nosocomial infections are evaluated and controlled and also findings of the study showed that local antibiotic prescription policies should be regularly monitored. Therefore, to better infection control, it is important to design programs such as controlling infections in different wards of hospitals, especially the intensive care unit, and antimicrobial resistance patterns should be monitored periodically in different regions.

It is also better in each hospital, using molecular methods such as ERIC-PCR, these bacteria are identified and their antibiotic resistance characteristics are determined and appropriate antibiotics are prescribed accordingly. Describing the relationship between clusters and genetic diversity in bacteria requires more extensive molecular studies.

## Conclusions

The choice of a genotyping technique depends on the skill level of the users and the facilities of the laboratory as well as the purpose of the study. In the present study, the ERIC-PCR technique was used to genetically classify *A. baumannii* strains as one of the most important and common microorganisms in nosocomial infections. Overall, the findings of the present study showed that the ERIC-PCR method is a simple, fast, and low-cost method for describing the genetic diversity of different strains of *A. baumannii.*

However, it is recommended that more studies be performed on samples taken from different hospitals across the country and that the ERIC-PCR method be compared with newer molecular methods such as PFGE.

## Data Availability

The datasets used and analyzed during the current study are available from the corresponding author on reasonable request.

## Abbreviations

ERIC-PCR: Enterobacterial Repetitive Intergenic Consensus-Polymerase chain reaction
MDR: multidrug-resistant
MBL: Metallo-β-lactamase
MLST: Multilocus sequence typing
PFGE: Pulse Field Gel Electrophoresis
